# The Effects of Switch Therapy in Osteoporosis Treatment after Romosozumab after Comparing with Prior Treatment

**DOI:** 10.1155/2024/2144527

**Published:** 2024-01-09

**Authors:** Akira Horikawa, Yuji Kasukawa, Michio Hongo, Akihisa Sano, Naohisa Miyakoshi

**Affiliations:** ^1^Shizuoka Tokushukai Hospital, 1-11 Surugaku-Simokawahara-Minami, Shizuoka 421 0117, Japan; ^2^Department of Orthopedic Surgery, Akita University Graduate School of Medicine, 1-1-1 Hondo, Akita 010-8543, Japan

## Abstract

*Rationale*. Although romosozumab is one of the most effective treatments for osteoporosis by increasing bone mineral density in the lumbar spine and femur and recommended for denosumab as switch therapy, these effects regarding its prior treatment have not yet been evaluated clearly. This study focused on the effects of switch therapy from romosozumab to denosumab in regard to prior treatment of osteoporosis including bone mineral density and bone turnover marker and other related factors. *Patient Concerns*. 15 osteoporotic patients were assigned to the naïve group, 15 were assigned to the teriparatide group, and 10 were assigned to the bisphosphonate group. *Interventions*. Patients who were treated as outpatients for osteoporosis with romosozumab for 1 year and switched to denosumab between 2020 and 2022 at our hospital were examined. Our hospital registry included 40 osteoporotic patients who were over 65 years of age with bone mineral density (bone mineral density): T score <−2.5 standard deviations (SDs) and fracture assessment tool (FRAX) score >20%. *Outcomes*. The naïve group had the highest increase in LS BMD among these three groups during switch therapy from romosozumab to denosumab, while there were no significant differences about adverse drug events and serum Ca concentration among them. There was no incidence of fracture. *Conclusion*. These findings indicate that the effects of osteoporotic treatment of switch therapy from romosozumab to denosumab were likely to affect prior treatment of osteoporosis.

## 1. Introduction

Recently, many kinds of anti-osteoporosis drugs have been developed to improve bone fragility through antibone resorptive (e.g., bisphosphonate) or bone anabolic effects (e.g., teriparatide) [1], and romosozumab appears to be used as the new choice for treating osteoporosis, including the treatment of vertebral fractures, in Japanese centers. Despite the recognized efficacy of romosozumab with respect to BMD, there are few reports of switch therapy after romosozumab. Therefore, we focused on the effects of switch therapy from romosozumab to denosumab in regard to prior treatment of osteoporosis including bone mineral density and bone turnover marker and other related factors.

## 2. Methods

### 2.1. Study Design

Patients who were treated as outpatients for osteoporosis with romosozumab for 1 year and switched to denosumab between 2020 and 2022 at our hospital were examined. Patients who were selected for this treatment were delegated with decision making by themselves and their families after explanation for all kinds of osteoporosis treatments at the initial phase of romosozumab. They were divided into three groups according to their prior treatment of osteoporosis: no past history of treatment (naïve, *n* = 15), anabolic treatment (teriparatide, *n* = 15), and antiabsorptive treatment (bisphosphonate, *n* = 10). Patients for the cohort were selected based on the standard for the diagnosis of osteoporosis: BMD (T-score < −2.5 SDs) and fracture assessment tool (FRAX) score >20%. All eligible patients were over 65 years of age. For the surveillance study, subjects were asked to identify fractures by taking plain radiographs of thoracic and lumbar spine on lateral views at baseline, 6 months, and 12 months after starting romosozumab and at 6 to 12 months after they switched to denosumab and the adverse drug events they had experienced during this treatment. To prevent hypocalcemia, all patients were prescribed Edirol (Chugai-Pharmaceutical Co., LTD., Japan) which includes active vitamin D3 formulation (active vitamin D 300 IU per 1 tablet) at the time of starting romosozumab. This study was conducted according to the Helsinki Declaration, and the medical ethics committee of Akita University Graduate School of Medicine approved this study (approval number: 1970). Written, informed consent was obtained from all participants.

### 2.2. Bone Mineral Density (BMD) Measurement

BMD was measured at the proximal femur (femoral neck) and lumbar spine (anteroposterior, L2-4) using dual-energy X-ray absorptiometry (DXA, Hologic QDR Discovery W type; Toyo Medic, Tokyo, Japan) at baseline, 6 months, 12 months after starting romosozumab, and 18 months after switching to denosumab during this trial.

### 2.3. Serum Biochemical Test Items

TRACP-5b (TRACP-5b ELISA, MBL, Nagoya, Japan), P1NP (P1NP ELISA, MBL), Ca, and eGFR were measured at above-mentioned phase. 25-hydroxyvitamin D (25-OHD) (ECLIA, BML, Tokyo, Japan) were also measured at baseline.

### 2.4. Statistical Analysis

Statistical analysis was performed using Microsoft Office Excel and the Statcel 4 program (OMS, Inc., Hyogo, Japan). Each subject such as Ca and eGFR was analyzed by the analysis of variance (ANOVA) to compare differences between the groups in the baseline. The differences were performed by Turkey test between naïve and teriparatide or naïve and bisphosphonate. The changes of BMD and bone turnover markers in each group were also analyzed by repeated measures analysis of variance with Turkey test. The All results of statistical tests were regarded as significant with *p* < 0.05.

## 3. Results

**Figure 1:**
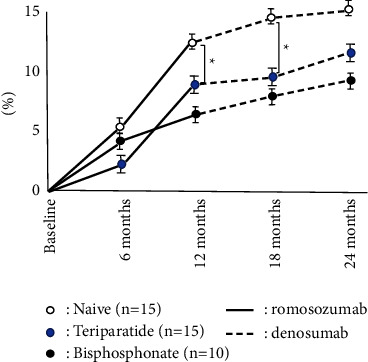
Chronological changes of BMD in the lumbar spine. ^*∗*^: *p* < 0.05: between groups, mean ± SD. The naïve group was highest increase in BMD compared among these three groups, and there were significant differences during romosozumab therapy and after switching denosumab.

**Figure 2:**
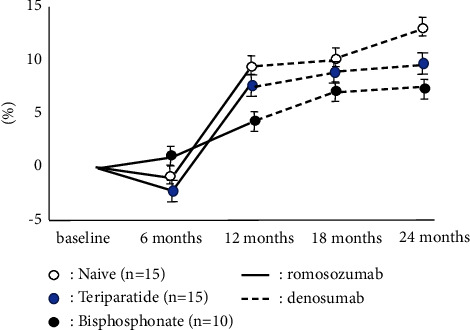
Chronological changes of BMD in the femoral neck. The change of BMD in femur had the tendency that naïve group was relatively higher than other groups as same as the change of BMD in lumbar spine although there were no significant differences among them.

**Figure 3:**
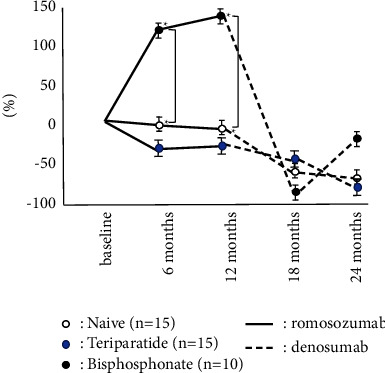
Chronological changes of P1NP. ^*∗*^: *p* < 0.05: between groups, mean ± SD. The change of P1NP in bisphosphonate group was significantly higher than other groups at every 6 and 12 months while there showed no significant differences in 18 months after denosumab.

**Figure 4:**
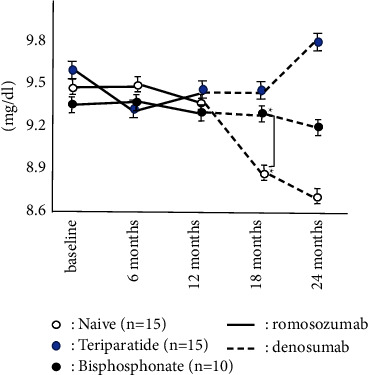
Chronological changes of serum Ca. ^*∗*^: *p* < 0.05: between groups, mean ± SD. The concentration of serum Ca showed significant differences in naïve group at 18 months after denosumab.

**Figure 5:**
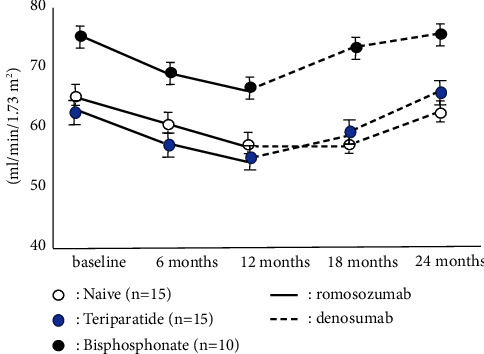
Chronological changes of eGFR. There was no significant difference in eGFR among all these groups.

**Figure 6:**
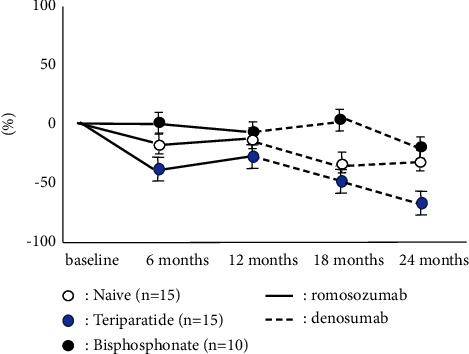
Chronological changes of TRACP-5b. There was no significant difference in TRACP-5b among all these groups.

The accumulated number of patients was 40 in all groups. Bone turnover marker was significantly high in the teriparatide group, while BMD and 25OHD in the teriparatide group were significantly lower than these groups on the baseline. There were no significant differences in BMI, Ca, and eGFR among groups (Table 1). The naïve group had the highest increase in LS BMD among these three groups, and there were significant differences during romosozumab therapy and after switching to denosumab. While teriparatide groups showed the second highest increase in LS BMD during romosozumab therapy, the bisphosphonate group showed the second highest increase in LS BMD after switching to denosumab (Figure 1). The change of BMD in the femur showed a similar tendency of the change of BMD in the lumbar spine although there were no significant differences among them (Figure 2). The change of P1NP in the bisphosphonate group was significantly higher than that of other groups at every 6 and 12 months while there were no significant differences in 18 months after denosumab (Figure 3). The concentration of serum Ca showed significant differences in the naïve group at 18 months after denosumab (Figure 4). There was no incidence of fracture and there were no significant differences in eGFR and TRACP-5b among all these groups (Figures 5 and 6).

## 4. Discussion

Although romosozumab has been established as the new and most effective treatment of osteoporosis [2–4] and there are few reports about the effects of treating osteoporosis by romosozumab in practice [5, 6], we did not find the reports about switch therapy after romosozumab to the best of our knowledge. Therefore, we focused on the effects of switching therapy after romosozumab by inducing denosumab because of its RANKL effects which are linked with Wnt signal pathway [7–9]. Similar to other reports on romosozumab, we found that the switch therapy will be needed to maintain bone mineral density after romosozumab [10].

Besides, there were a few reports about the treatment of osteoporosis by romosozumab by comparing prior treatment of osteoporosis [5, 6]; our study indicated that switch therapy after romosozumab was similar to the primary treatment of romosozumab. In regard to this point, Tominaga also suggested that the change of increase in BMD were naïve>teriparatide> bisphosphonate>denosumab in 6 months [6]. Moreover, Ebina also suggested that naïve, teriparatide, bisphosphonates and denosumab had higher increase of BMD in turn [5]. In addition, Langdahl also reported that romosozumab is very effective in treating naïve patients [11]. Considering these reports and our current results, naïve is the most effective among these prior therapies after switching therapy from romosozumab to denosumab because its bone turnover status is normal that seems to be most sensitive to the treatment of osteoporosis.

Although this study has limitations and bias, including other kinds of anti-osteoporosis medications and the number and duration was small, further investigations including trials of these medications will be needed in the near future.

In summary, the effects of osteoporotic treatment of switch therapy from romosozumab to denosumab were likely to affect prior treatments of osteoporosis. This treatment will be efficient for any kind of prior treatment of osteoporosis.

## 5. Conclusion

The effects of treatment for switching therapy of romosozumab were examined. The results showed that naïve group was most effective by switch therapy from romosozumab to denosumab in the change of bone mineral density, while there were no significant differences about adverse drug events and serum Ca concentration among them. There was no incidence of fracture.

## Figures and Tables

**Table 1:** Baseline characteristics by group.

	Naïve group	Teriparatide group	Bisphosphonate group	*p* value
Number	15	15	10	
Age (y)	80.9 ± 2.3	80.0 ± 8.4	74.1 ± 6.4	0.055
BMI (kg/m^2^)	25.5 ± 6.3	24.9 ± 5.8	24.1 ± 3.8	0.301
BMD: femur (g/cm^2^)	0.519 ± 0.150	0.441 ± 0.102^a^	0.511 ± 0.001	<0.05^a^
BMD: lumbar spine (g/cm^2^)	0.709 ± 0.095	0.648 ± 0.144^a^	0.693 ± 0.045	<0.05^a^
Serum calcium (g/dl)	9.5 ± 0.4	9.6 ± 0.5	9.3 ± 0.3	0.097
eGFR (ml/min/1.73 m^2^)	64.3 ± 18.7	62.4 ± 20.1	76.1 ± 12.9	0.053
P1NP (ng/ml)	53.2 ± 18.5	77.5 ± 19.1	16.7 ± 5.4^a^	<0.05^a^
TRACP-5b (mU/dl)	321.5 ± 116.6	463.0 ± 111.6	211.7 ± 56.9^a^	<0.05^a^
25OHD (ng/ml)	11.7 ± 6.5	10.0 ± 5.6^a^	16.5 ± 5.3	<0.05^a^
Previous fracture	None	None	None	

Values are expressed as means and standard deviation. ^a^ANOVA. BMI: body mass index, BMD: bone mineral density, eGFR: estimated glomerular filtration rate, P1NP: procollagen 1 N-terminal propeptide, TRACP-5b: tartrate-resistant acid phosphatase 5b, and 25OHD: 25-hydroxyvitamin D.

## Data Availability

The data used to support the findings of this study are restricted by the medical ethics committee of Akita University Graduate School of Medicine in order to protect patient privacy. Data are available from Naohisa Miyakoshi (miyakosh@doc.med.akita-u.ac.jp) for researchers who meet the criteria for access to confidential data.

## Abbreviations

BMD: Bone mineral density
FRAX: Fracture assessment tool
25-OHD: 25-hydroxyvitamin D
DXA: Dual-energy X-ray absorptiometry
TRACP-5b: Tartrate-resistant acid phosphatase
P1NP: Type 1 procollagen N-terminal propeptide
eGFR: Estimated glomerular filtration
RANKL: Receptor activator of nuclear factor-kappa B ligand.
